# The LINC Between Mechanical Forces and Chromatin

**DOI:** 10.3389/fphys.2021.710809

**Published:** 2021-08-02

**Authors:** Olga Lityagina, Gergana Dobreva

**Affiliations:** ^1^Medical Faculty Mannheim, Heidelberg University, Mannheim, Germany; ^2^German Centre for Cardiovascular Research (DZHK), Partner Site Heidelberg/Mannheim, Mannheim, Germany

**Keywords:** LINC complex, nuclear lamins, mechanotransduction, epigenetics, cardiovascular disease, cardiomyocyte, endothelial cell

## Abstract

The heart continually senses and responds to mechanical stimuli that balance cardiac structure and activity. Tensile forces, compressive forces, and shear stress are sensed by the different cardiac cell types and converted into signals instructing proper heart morphogenesis, postnatal growth, and function. Defects in mechanotransduction, the ability of cells to convert mechanical stimuli into biochemical signals, are implicated in cardiovascular disease development and progression. In this review, we summarize the current knowledge on how mechanical forces are transduced to chromatin through the tensed actomyosin cytoskeleton, the linker of nucleoskeleton and cytoskeleton (LINC) complex and the nuclear lamina. We also discuss the functional significance of the LINC complex in cardiovascular disease.

## Introduction

Mechanical forces play a key role in the development, maturation and function of the heart. During heart formation, contractions of cardiomyocytes (CMs) cause blood to flow over the cardiac endothelial lining, which leads to generation of mechanical cues such as shear stress and cyclic strain that further aid and guide cardiac morphogenesis (Granados-Riveron and Brook, 2012). After birth, these forces instruct and maintain the healthy heart functional state (Andrés-Delgado and Mercader, 2016). The type and magnitude of mechanical forces, such as shear stress, cyclic stretch, and alterations in the extracellular matrix (ECM) stiffness, have to be faithfully recognized from the different cardiac cell types to allow their adaptation to the dynamic changes of their surrounding by modifying gene expression. The inability of cells to correctly translate mechanical cues into biochemical signals, caused by mutations or deregulation of proteins that disturb mechanosensing and mechanotransduction, can contribute to the development and progression of cardiovascular diseases (Jaalouk and Lammerding, 2009).

**FIGURE 1:**
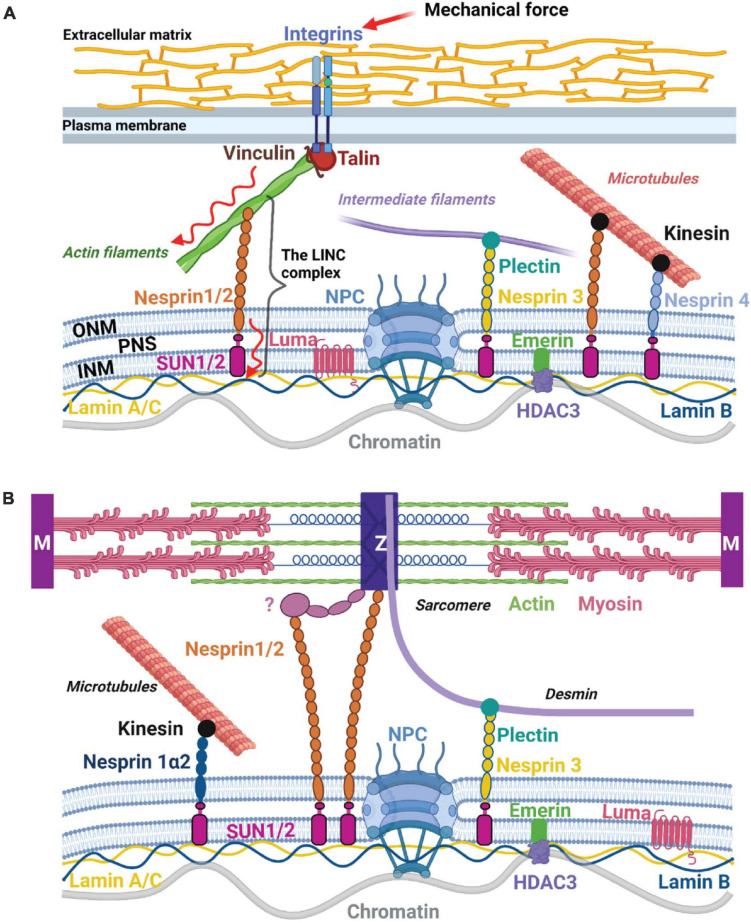
The LINC complex and nuclear mechanotransduction. **(A)** Composition of the LINC complex and nuclear mechanotransduction. The LINC complex consists of KASH-domain containing proteins (Nesprins) located in the outer nuclear membrane (ONM) and SUN-domain proteins located in the inner nuclear membrane (INM). Nesprins bind to different cytoskeletal components in the cytoplasm and to the SUN-domain proteins in the perinuclear space (PNS). On the other side, SUN proteins interact with the nuclear lamins and other inner nuclear membrane-associated proteins such as Emerin and Luma (TMEM43). The nuclear lamina, in turn, anchors chromatin to the nuclear periphery. LINC complex composition varies in different cell types. Mechanical forces (increase in ECM matrix stiffness, shear stress, and stretch) sensed by the mechanosensitive cell-surface receptors and transduced via the cytoskeleton and the LINC complex to the nuclear lamina may directly influence chromatin architecture and gene expression. **(B)** The CM LINC complex. In CMs Nesprins-1/2 connect to the Z-disk (Z) – directly or indirectly through other proteins indicated with a question mark, while Nesprin-3 binds to Desmin through Plectin. Figures were created with BioRender.com and are not to scale.

The role of cytosolic signaling pathways and mechanosensitive transcription factors in mediating cellular responses to mechanical forces has been long recognized and extensively studied. Mechanosensitive ion channels and transmembrane receptors, as well as cytoskeleton and sarcomeric proteins have been shown to activate signaling cascades [e.g., through Rho GTPases, MAPKs, phospholipase C, calcium/calcineurin, focal adhesion kinase (FAK), Src, integrin-linked kinase (ILK) etc.], which converge into the nucleus to induce transcriptional programming that dictates cell behavior and function (Hahn and Schwartz, 2009; Jaalouk and Lammerding, 2009; Wang et al., 2009). For example, active Rho-GTPase signaling and actomyosin-mediated contractility result in the translocation of mechanosensitive transcription factors MRTFA (MKL1) and YAP/TAZ from the cytoplasm to the nucleus, where they initiate specific transcriptional programs (Dupont et al., 2011; Dorn et al., 2018). While nucleocytoplasmic shuttling of transcriptional regulators to mediate responses elicited by mechanical stimuli has been extensively studied, more recent work has suggested a role for the nucleus in direct propagation of mechanical stress via the linker of nucleoskeleton and cytoskeleton (LINC) complex, a process referred to as nuclear mechanotransduction (Wang et al., 2009; Kirby and Lammerding, 2018). The LINC complex consists of KASH-domain spectrin repeat proteins (Nesprins), located in the outer nuclear membrane and SUN (Sad1 and UNC84)-domain transmembrane proteins, located in the inner nuclear membrane (Figure 1A). Nesprins bind to cytoskeletal elements such as microtubuli, intermediate filaments and actin in the cytoplasm and to the SUN proteins in the perinuclear space (Isermann and Lammerding, 2013). In CMs, nesprins can connect directly to the Z-disk or indirectly through other proteins (Stroud et al., 2014; Figure 1B). Inside the nucleus, SUN proteins bind to lamin A, which, in turn, anchors chromatin to the nuclear lamina. The nuclear lamina consists of A-type (lamin A and C) and B-type lamins (lamin B1 and B2), which form distinct meshworks (Shimi et al., 2008). While lamins B1 and B2 are localized at the periphery and associate mainly with transcriptionally silent chromatin (Reddy et al., 2008; Wen et al., 2009), lamins A and C are found at the nuclear periphery as well as in the nuclear interior and associate with both hetero- and transcriptionally active euchromatin (Gesson et al., 2016). However, three-dimensional structured illumination microscopy analysis of lamin meshworks in HeLa cells showed that the loss of A-type lamins results in alterations in B-type meshworks and *vice versa* (Shimi et al., 2008), suggesting that their activity might be interconnected and that mechanosensitive mechanisms could affect both lamin A and lamin B lamina-associated chromatin domains (LADs). A few other proteins, including Emerin, Luma (TMEM43) and LAP2α have been shown to interact with the LINC complex components (Stroud, 2018). In this review, we summarize the current knowledge on the mechanisms of a direct mechanical force propagation to chromatin through the tensed actomyosin cytoskeleton, the LINC complex and the nuclear lamina and their malfunction in cardiovascular diseases.

## Force Transmission to the Nucleus

**FIGURE 2:**
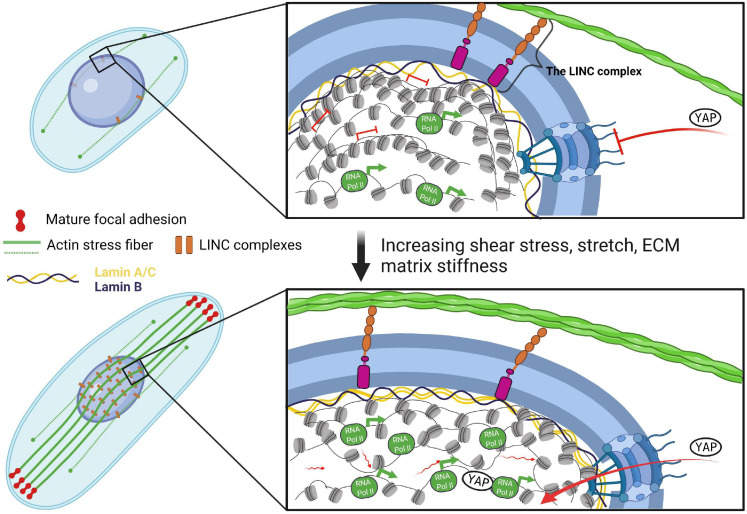
Direct mechanical force transmission to chromatin through the LINC complex. Increasing mechanical forces lead to the formation of apical actin stress fibers, which propagate these cues to the nucleus via the LINC complex and cause its flattening and elongation. Nuclear deformation results in quantitative and conformational changes in the nuclear lamina, leading to alterations in chromatin fluidity and architecture coupled to changes in RNA Pol II occupancy and gene transcription. Additionally, nuclear pores are stretched as a consequence of nuclear elongation, which enables YAP translocation through decreased mechanical restriction of nuclear pore-mediated molecular transport. Tension-induced epigenetic and transcriptional response feeds back to the cytoskeleton and the extracellular matrix to balance outside and inside forces.

The connection between the ECM and the cell cytoskeleton is established through dynamic, integrin-containing multi-protein complexes that simultaneously bind to ECM proteins and anchor actin filaments, the focal adhesions (Figures 1, 2). In response to tensile stress, as modeled by culturing mouse embryonic fibroblasts (MEF) on large and elongated micropatterns, cells establish strong connections to the substrate (mature focal adhesions) at the two opposite poles along the long axis of the cell, which leads to the formation of actin stress fibers along the cell’s long axis in the apical plane (Alisafaei et al., 2019). Actin stress fibers are contractile cytoskeletal structures, composed mainly from actin and non-muscle myosin II (actomyosin). Stress fibers then propagate tensile forces from the mature focal adhesions to the nucleus (Alisafaei et al., 2019; Figures 1, 2). Local shear stress, as modeled through the movement of a magnetic bead attached to the cell membrane of Chinese hamster ovary (CHO) cells is also propagated to the nucleus in an actomyosin-dependent fashion via focal adhesions (Tajik et al., 2016). However, forces that are applied on the cell membrane, but bypass the focal adhesions, can also be transmitted via actin, albeit less effectively compared to the force propagation from focal adhesions (Tajik et al., 2016). Interestingly, prolonged stretching of MEFs induces thickening of discrete actin stress fibers coupled to dense accumulation of lamin A/C in the apical side of the nucleus and the formation of lamin A/C dents along the individual actin fibers (Kim et al., 2017). These indentation sites are characterized by a local enrichment of LINC complexes, which connect the stress fibers to the nuclear lamina, as shown in endothelial cells (Versaevel et al., 2015). Isolated nuclei alone are also able to respond to tension force applied directly via Nesprin-1 (Guilluy et al., 2014), demonstrating that the LINC complex is crucial for the force transmission into the nucleus.

## Influence of Mechanical Forces on Nuclear Morphology, Chromatin Organization, and Gene Transcription

Recent studies using different models have greatly expanded our understanding of the effects of mechanical forces on nuclear morphology and chromatin organization and the role of the actomyosin meshworks in force transmission (Figures 1, 2). For example, tensile forces arising from culturing fibroblasts on fibronectin-coated micropatterns to mimic the *in vivo* microenvironment have been shown to trigger an actomyosin-dependent alterations in nuclear morphology, lamin A/C levels and shuttling of epigenetic factors (Alisafaei et al., 2019). In a similar way, 1 h cyclic stretch (1 Hz, 8% uniaxial cyclic stretch within the physiological range) alters the nucleus shape, which becomes flattened (approx. 35% reduction in nuclear height) and slightly elongated in the direction of stretch (Kim et al., 2017). Interestingly, tensile force-induced nuclear flattening stretches the nuclear pores, which decreases the mechanical restriction of nuclear pores-mediated molecular transport and results in accumulation of the mechanosensitive transcription factor YAP in the nucleus (Elosegui-Artola et al., 2017; Figure 2), providing an interconnection between the cytosolic pathways-mediated response of cells to mechanical forces and direct force transmission to the nucleus through the actomyosin cytoskeleton and the LINC complex. Further, stretch of the nuclear envelope in response to tensile stress results in increased lamin A/C levels (Swift et al., 2013; Alisafaei et al., 2019) and decreased mobility through inhibition of lamin A/C Ser22 phosphorylation, which regulates lamin A/C turnover and physical properties, as shown in mesenchymal stem cells (Buxboim et al., 2014). In addition, as a result of compressive forces on nuclei the nuclear lamina acquires structurally polarized state, in which epitopes at the N- and C-terminus of lamins A/C at the basal nuclear envelope become inaccessible (Ihalainen et al., 2015). Recent studies have demonstrated that lamins anchor LADs at the nuclear periphery and are responsible for maintaining proper interactions among topologically associated chromatin domains (TADs), as well as for the maintaining of active and inactive chromatin and transcriptional states (Zheng et al., 2018). Importantly, loss of chromatin tethering to the nuclear lamina following HDAC3 deletion causes release of CM-specific gene regions from the nuclear periphery, leading to precocious CM differentiation and heart disease pathogenesis (Poleshko et al., 2017). Ablation of CTCF, an architectural protein enriched at LAD and TAD boundaries that binds DNA and facilitates chromatin looping, in cardiomyocytes results in cardiomyopathy (Rosa-Garrido et al., 2017). Thus, mechanical forces-induced conformational changes in the nuclear lamina might have a profound effect on chromatin organization, chromatin dynamics and gene expression in cardiovascular cells.

Several studies suggested that both chromatin organization and nuclear lamins have an impact on nuclear stiffening and might work synergistically to maintain nuclear rigidity in response to mechanical stress. Indeed, chromatin decondensation decreases while lamin A overexpression increases the anisotropic nuclear deformations caused by constant force application on fibroblast nuclei using a pyramidal atomic force microscope (AFM) tip (Haase et al., 2016). A more recent study using isolated nuclei from HeLa cells demonstrated that in nuclei stretched at a physiologically relevant speed of 50 nm/s, lamin A/C levels modulate the nuclear stiffness primarily in response to more than 3 μm of stretch, whereas euchromatin/heterochromatin levels mainly control nuclear stiffening at less than 3 μm stretch (Stephens et al., 2017). Moreover, disruption of the LINC complex abrogates the difference between heterochromatin and euchromatin elasticities/stiffnesses at the peak nuclear deformation induced by CM contraction (Ghosh et al., 2021), suggesting that an intact LINC complex is also necessary for maintaining chromatin stiffness. Chromatin decondensation further results in an increased movement of chromatin, while inhibition of myosin II and decoupling the nucleus from the cytoskeleton decreases chromatin mobility in HUVEC cells (Spagnol and Noel Dahl, 2014), further supporting the notion that the intact actomyosin apparatus and the LINC complex are critical factors for the mechanical signal transmission to chromatin. Accordingly, an increase in euchromatin formation and/or heterochromatin depletion leads to decreased nuclear rigidity (softer nuclei) and formation of nuclear blebs in fibroblasts (Furusawa et al., 2015; Stephens et al., 2018). In turn, nuclear blebbing can be counteracted through extracellular multivalent cation transduction, which simulates the transient calcium (Ca^2+^) influx associated with activation of mechanosensitive channels and increases heterochromatin formation (Stephens et al., 2019). Intriguingly, a recent study utilizing skin epidermis stem/progenitor cells reported Ca^2+^-dependent rapid nuclear response preventing DNA damage upon nuclear deformation evoked by cyclic stretch, followed by a long-term adaptation mechanisms modulating chromatin organization (Nava et al., 2020). In the rapid cell response to mechanical stress, Ca^2+^ released from the endoplasmic reticulum (ER) reduces lamina-associated heterochromatin, thereby diminishing nuclear membrane tension and increasing chromatin fluidity to prevent DNA breaks. Subsequently, nuclear strain is hampered by cell-cell contacts-mediated cytoskeleton reorganization and cell and nuclear reorientation along the long axis, allowing chromatin adaptation for long-term mechanoprotection. On the other hand, Ca^2+^ release from the ER is dependent on lamin A levels and nuclear stiffness (Nava et al., 2020), suggesting that cytosolic and nuclear mechanotransduction pathways are tightly intertwined through both positive and negative feedbacks. Consistently, acute modulation of contractility and ECM elasticity of embryonic hearts resulted in rapid and reversible changes in lamin A levels, DNA damage as well as cell cycle and loss of function experiments revealed a key role of lamin A in safeguarding against DNA-damage and cell cycle arrest of cardiomyocytes subjected to mechanical stress (Cho et al., 2019).

As discussed above, myriad studies have demonstrated the critical role of the LINC complex and lamin A in nuclear and chromatin reorganization as well as preventing DNA damage upon biophysical forces. On the other hand, chromatin structure regulates gene transcription by determining the accessibility of the transcriptional machinery to DNA, implying that changes in chromatin organization in response to mechanical stimuli could have a direct impact on gene transcription. Indeed, forces applied on the CHO cell surface via integrin-bound Arg-Gly-Asp-coated magnetic beads stretch chromatin and instantly upregulate gene transcription (Tajik et al., 2016). Specifically, using GFP tagged bacterial-chromosome dihydrofolate reductase (DHFR) transgene, Tajik et al. (2016) demonstrated that mechanical forces propagated to chromatin via the actomyosin and the LINC complex induce chromatin stretching coupled to transcriptional upregulation of DHFR. Further study from the same group revealed that the direction of force application and the stress amplitude also influence the extent of chromatin fluidity and gene expression (Wei et al., 2020). Although these conclusions were based on the transcriptional changes of the artificial DHFR transgene, a recent study suggested that the endogenous genes *EGR1* and *CAV1* respond similarly to directional mechanical stimulation (Sun et al., 2020). Moreover, force-induced gene upregulation depends on the levels and the spatial location of H3K9me3 histone mark, i.e., force-induced demethylation of H3K9me3 in the nuclear interior, but not near the nuclear periphery, leads to Pol II recruitment to promoters of mechanoresponsive genes to rapidly upregulate their transcription (Sun et al., 2020). Similarly, short-term stretch (30 min) of epidermal progenitor cells resulted in decrease of H3K9me3 (Nava et al., 2020), while long-term stretch resulted in decrease in global transcription due to increased H3K27me3-occupancy resulting in silencing of epidermal differentiation genes (Le et al., 2016; Nava et al., 2020), suggesting different modes of epigenetic response to short- and long-term stretch. In the heart, increased mechanical stress induced through TAC surgery, also leads to chromatin reorganization and higher transcriptional activity through increased recruitment of RNA polymerase II in CMs *in vivo*, which is dependent on the spatial location of genes in the nucleus (Karbassi et al., 2019). While duration and magnitude of mechanical stress application proportionally upregulates gene transcription (Tajik et al., 2016; Wei et al., 2020) and could play an important role in cell adaptation, too long lasting stress could induce non-reversible adaptations, which, in turn, might lead to disease development or contribute to disease progression.

## The LINC Complex in Cardiovascular Disease

Consistent with the important biological functions of the LINC complex in mechanical stress response, mutations in different components of the LINC complex and its interaction partners (Nesprin-1 and Nesprin-2, lamin A, Emerin, and TMEM43) are associated with diseases of the heart and striated muscles that experience high levels of mechanical stress, including dilated cardiomyopathy (DCM), arrhythmogenic cardiomyopathy (ACM), and Emery-Dreifuss muscular dystrophy (EDMD) (Stroud et al., 2014). One of the most frequently mutated genes associated with familial DCM is Lamin A/C (*LMNA*) (Hershberger et al., 2013). Cheedipudi et al. (2019) found redistribution of LADs in hearts of DCM patients carrying pathogenic *LMNA*-mutations, which correlated with CpG methylation and gene expression changes. In addition, abnormal binding of lamin A or LAP2α-lamin A/C complexes to euchromatin and dysregulation of the WNT/β-catenin and TGFβ-BMP pathways might also contribute to the disease phenotype (Zhang et al., 2021). Further evidence that *LMNA* mutations disrupt lamina-chromatin interactions and influence gene expression comes from *in vitro* experiments using human induced pluripotent stem cells (hiPSCs)-derived CMs. HiPSCs-CMs harboring DCM-associated T10I and R541C *LMNA* mutations exhibit specific alterations in the peripheral chromatin, resulting in an increased and aberrant expression of non-myocyte lineage genes (Shah et al., 2021). Taken together, these studies support a key role of lamin A/C in chromatin organization for proper cardiac function.

Dilated cardiomyopathy development and progression is often associated with altered Ca^2+^ handling in cardiac myocytes. Indeed, *Lmna* H222P mutation results in an abnormal increase of sarcolipin, an inhibitor of the sarco/endoplasmic reticulum (SR) Ca^2+^ ATPase (SERCA) in mouse ventricular CMs, leading to altered calcium handling already in the early stage of DCM, before changes in left ventricular function have occurred (Morales Rodriguez et al., 2020). Further, age-dependent biochemical remodeling of the ryanodine receptor 2 (RYR2) in the heart that lead to “leaky” RYRs and a subsequent increase of the SR Ca^2+^ leak has been shown to play a role in cardiomyopathy caused by *LMNA* H222P mutation (Dridi et al., 2021). A recent study showed that iPSC-CMs harboring K117fs *LMNA* mutation, which ultimately causes *LMNA* haploinsufficiency, display aberrant calcium homeostasis that leads to arrhythmias (Lee et al., 2019). Mechanistically, Lee et al. (2019) demonstrated that abnormal epigenetic activation of *PDGFRB*, caused by the K117fs *LMNA* mutation, resulted in an increased CAMK2D and RYR2 phosphorylation and arrhythmias. To what extend changes in Ca^2+^ levels resulting from RYR2 phosphorylation induces further changes in chromatin structure in *LMNA* mutant CMs still needs to be elucidated. Intriguingly, epigenetic silencing of *SCN5A* caused by a different *LMNA* mutation, K219T, leads to decreased expression of Na_*v*_1.5 channel, altered action potential, reduced peak sodium current and diminished conduction velocity in iPSC-CMs (Salvarani et al., 2019), further highlighting the role of lamin A in epigenetic regulation of ion homeostasis in CMs. However, more studies are required to elucidate the common and distinct mechanisms caused by different pathogenic *LMNA* mutations on CM ion handling and triggered arrhythmias.

Since CMs represent the main functional unit of the heart, most studies investigating the development and progression of cardiac diseases associated with *LMNA* mutations have been focusing on this cell type. However, within the heart, CMs, endothelial cells (ECs) and fibroblasts (FBs) all sense and respond to mechanical stimuli and communicate, thereby affecting each other’s behavior and functionality (Tirziu et al., 2010; Granados-Riveron and Brook, 2012; Tian and Morrisey, 2012; Saucerman et al., 2019). Up to now, only few studies examined the EC contribution to cardiac laminopathies (Osmanagic-Myers et al., 2018; Sayed et al., 2020). Sayed et al. (2020) showed that *LMNA* K117fs mutation causes epigenetic silencing of Krüppel-like factor 2 (KLF2) in hiPSCs–derived ECs, which in turn leads to impaired KLF2-mediated EC response to shear stress and EC dysfunction. Impaired shear stress response has also been noted in endothelium-specific progeria mouse model (Osmanagic-Myers et al., 2018). Endothelial specific progerin expression affected the levels and structural organization of actin as well as nuclear envelope proteins involved in shear stress force transmission, namely SUN1/2 and Emerin. Although cardiac FBs play an important role in ECM homeostasis, the distribution of mechanical forces through the cardiac tissue, but also in adverse cardiac remodeling after myocardial infarction, the role of lamin A loss in fibroblasts in cardiac disease development is not known. Thus, understanding the fibroblast contribution and the heterocellular crosstalk in cardiac laminopathies will be instrumental for the discovery and design and novel therapeutic strategies for this life-threatening disease.

Alterations in other members of the LINC complex also play an instrumental role in cardiac disease pathogenesis. For example, SUN2-null mice display cardiac hypertrophy with concomitant increase in AKT/MAPK signaling, similar to mice lacking A-type lamins, but do not develop fibrosis or upregulate pathological hypertrophy markers, in contrast to lamin A/C-null mice (Stewart et al., 2019). Haploinsufficiency of *TMEM43* gene specifically in CMs leads to a late-onset cardiomyopathy accompanied by myocardial fibrosis (Rouhi et al., 2020). Nesprins play an important role in the protection of CMs against mechanical stress-induced pathophysiological changes. Depletion of Nesprin-3 or its binding partner desmin leads to a nucleus collapse, loss of genome organization, DNA damage and broad transcriptional changes that may contribute to the pathophysiological changes observed in desmin-related cardiomyopathies (Heffler et al., 2020), highlighting an important role of the desmin cytoskeleton in nuclear stability and genome organization. Dual ablation of both Nesprin 1 and 2 in CMs results in early onset cardiomyopathy with mutant CMs exhibiting altered nuclear positioning and shape as well as chromatin architecture (Banerjee et al., 2014). Moreover, several studies conducted in *Drosophila* have suggested that the LINC complex members are crucial for nuclear positioning-guided sarcomere formation (Auld and Folker, 2016; Wang et al., 2018). Muscle specific depletion of either the KASH domain–containing protein klarsicht (klar) or the SUN domain–containing protein klaroid (koi) blocked the recruitment of the Z-line protein ZASP to the nucleus during the early stages of sarcomere assembly, resulting in sarcomere formation defects (Auld and Folker, 2016). However, further investigations need to be conducted to address whether nuclear positioning precedes sarcomere formation in the mammalian heart. Nesprins might also be important in regulating ECs function upon mechanical forces-induced pathophysiological changes. In human umbilical vein ECs, knockdown of either Nesprin-1 or Nesprin-2 leads to an increase in EC spreading and stress fiber levels and decreases EC migration (King et al., 2014). Moreover, knockdown of Nesprin-3 attenuated the directional migration of human aortic ECs in response to shear stress (Morgan et al., 2011). Thus, understanding the role of the different components of the LINC complex in cardiac endothelial cells will be critical to understanding the mechanosensitive mechanisms in EC controlling cardiac function.

Taken together, these data demonstrate the importance of the LINC complex and its interaction partners for proper cardiovascular function and highlights the role of the LINC complex as a regulatory hub translating mechanical signals into chromatin changes.

## Concluding Remarks and Perspectives

The response of cells to mechanical cues is a complex multilevel process that allows cells to adapt to the changes in their microenvironment. Recent studies have brought into the spotlight the direct mechanical force propagation to chromatin and the molecular players involved in this process, such as the actomyosin, the LINC complex and the nuclear lamins. Tension-induced epigenetic and transcriptional programming in the distinct cardiovascular cell types feeds back to the cytoskeleton and the extracellular matrix to balance the outside and inside forces. Thus, dissecting the players at the interface of mechanical forces and epigenetics and understanding the heterocellular crosstalk in the heart evoked by biophysical stimuli will bring key insights into the mechanosensitive mechanisms underlying cardiovascular function and dysfunction, and pinpoint novel therapeutic targets for cardiovascular diseases, the leading cause of death globally.

## Author Contributions

Both authors listed have made a substantial, direct and intellectual contribution to the work, and approved it for publication.

## Conflict of Interest

The authors declare that the research was conducted in the absence of any commercial or financial relationships that could be construed as a potential conflict of interest.

## Publisher’s Note

All claims expressed in this article are solely those of the authors and do not necessarily represent those of their affiliated organizations, or those of the publisher, the editors and the reviewers. Any product that may be evaluated in this article, or claim that may be made by its manufacturer, is not guaranteed or endorsed by the publisher.
